# Association Between Alzheimer Disease and Cancer With Evaluation of Study Biases: A Systematic Review and Meta-analysis

**DOI:** 10.1001/jamanetworkopen.2020.25515

**Published:** 2020-11-13

**Authors:** Monica Ospina-Romero, M. Maria Glymour, Eleanor Hayes-Larson, Elizabeth Rose Mayeda, Rebecca E. Graff, Willa D. Brenowitz, Sarah F. Ackley, John S. Witte, Lindsay C. Kobayashi

**Affiliations:** 1 Department of Epidemiology and Biostatistics, University of California, San Francisco; 2 now at Department of Pathology and Laboratory Medicine, University of Wisconsin School of Medicine and Public Health, Madison; 3 Department of Epidemiology, Jonathan and Karin Fielding School of Public Health, University of California, Los Angeles; 4 Department of Psychiatry, University of California, San Francisco; 5 Department of Epidemiology, University of Michigan School of Public Health, Ann Arbor

## Abstract

**Importance:**

Observational studies consistently report inverse associations between cancer and Alzheimer disease (AD). Shared inverse etiological mechanisms might explain this phenomenon, but a systematic evaluation of methodological biases in existing studies is needed.

**Objectives:**

To systematically review and meta-analyze evidence on the association between cancer and subsequent AD, systematically identify potential methodological biases in studies, and estimate the influence of these biases on the estimated pooled association between cancer and AD.

**Data Sources:**

All-language publications were identified from PubMed, Embase, and PsycINFO databases through September 2, 2020.

**Study Selection:**

Longitudinal cohort studies and case-control studies on the risk of AD in older adults with a history of any cancer type, prostate cancer, breast cancer, colorectal cancer, or nonmelanoma skin cancer, relative to those with no cancer history.

**Data Extraction and Synthesis:**

Two reviewers independently abstracted the data and evaluated study biases related to confounding, diagnostic bias, competing risks, or survival bias. Random-effects meta-analysis was used to provide pooled estimates of the association between cancer and AD. Metaregressions were used to evaluate whether the observed pooled estimate could be attributable to each bias. The study was designed and conducted according to the Preferring Reporting Items for Systematic Reviews and Meta-analyses (PRISMA) reporting guideline.

**Main Outcomes and Measures:**

Incidence, hazard, or odds ratios for AD comparing older adults with vs without a previous cancer diagnosis.

**Results:**

In total, 19 cohort studies and 3 case-control studies of the associations between any cancer type (n = 13), prostate cancer (n = 5), breast cancer (n = 1), and nonmelanoma skin cancer (n = 3) with AD were identified, representing 9 630 435 individuals. In all studies combined, cancer was associated with decreased AD incidence (cohort studies: random-effects hazard ratio, 0.89; 95% CI, 0.79-1.00; case-control studies: random-effects odds ratio, 0.75; 95% CI, 0.61-0.93). Studies with insufficient or inappropriate confounder control or greater likelihood of AD diagnostic bias had mean hazard ratios closer to the null value, indicating that these biases could not explain the observed inverse association. Competing risks bias was rare. Studies with greater likelihood of survival bias had mean hazard ratios farther from the null value.

**Conclusions and Relevance:**

The weak inverse association between cancer and AD may reflect shared inverse etiological mechanisms or survival bias but is not likely attributable to diagnostic bias, competing risks bias, or insufficient or inappropriate control for potential confounding factors.

## Key Points

**Question:**

Does an association exist between cancer and subsequent Alzheimer disease (AD), and how likely is it that such a finding is associated with methodological bias rather than with a true common etiology?

**Findings:**

In this systematic review and meta-analysis of 22 cohort and case-control studies representing 9 630 435 individuals, cancer diagnosis was associated with 11% decreased incidence of AD. Bias-adjusted metaregressions suggested that competing risks and diagnostic bias were unlikely explanations for the observed association, whereas survival bias remains to be ruled out.

**Meaning:**

The observed inverse association between cancer and AD does not seem to be a consequence of competing risks, known confounding, or diagnostic bias.

## Teaser

This systematic review and meta-analysis assesses the likelihood that methodological biases in cohort studies and case-control studies, rather than a common etiology, explain the inverse association between cancer and subsequent Alzheimer disease incidence among older adults.

## Introduction

Cancer and Alzheimer disease (AD) and related dementias are consistently inversely associated in epidemiologic studies.^1,2^ This inverse association is counterintuitive because patients with a cancer diagnosis experience stress, treatment with cytotoxic drugs, invasive surgical procedures, and persistent pain, exposures that might decrease cognitive capacity and increase risk of developing AD relative to the cancer-free population of similar age.^3,4,5,6^ However, 2 meta-analyses of observational studies published up to 2014 reported an approximately 35% lower incidence of AD among older cancer survivors compared with those with no cancer history.^7,8^ This paradoxical inverse association has persisted in more recent studies that have implemented methodological approaches to reduce study biases that could induce this association.^9,10,11^

A compelling hypothesis for the inverse association between cancer and AD is a common etiology that acts in opposite directions in carcinogenesis and neurodegeneration.^12^ Proposed biological mechanisms involve proteins that suppress tau and amyloid-β deposition and regulate the cell cycle,^13,14^ common epigenetic modifications,^15^ and age-related dysregulation of cellular metabolism.^16^ If true, this potential common etiology represents a major opportunity to gain insight into the causes of both carcinogenesis and neurodegeneration.^12,17,18,19^

The inverse cancer-AD association could also be an artifact of methodological biases, such as bias related to handling of potential confounders, diagnostic bias, competing risks, or selective survival.^17,20^ The extent of these biases, or their effects on pooled risk estimates for the cancer-AD association, has not been systematically evaluated. Systematically combining evidence from studies with different biases (ie, evidence triangulation) may offer the most convincing interpretation of the literature.^21,22^ We thus conducted a systematic review and meta-analysis of existing literature on the association between cancer and subsequent AD risk. We evaluated the plausibility of each type of bias in each contributing study and used metaregressions to quantify the potential influence of each bias on the pooled risk estimate for the cancer-AD association.

## Methods

### Design and Search Strategy

The search included articles in any language published in the PubMed, Embase, and PsycINFO electronic databases through September 2, 2020. The search was conducted using the following keywords: *neoplasia or cancer or malignancy* and cognitive dysfunction or cognitive impairment or Alzheimer* and *epidemiologic study or cohort or case-control or longitudinal study* and *adult or middle age or elder*. This study was designed and conducted according to the Preferred Reporting Items for Systematic Reviews and Meta-analyses (PRISMA) reporting guideline.^23^

### Study Selection and Data Extraction

Inclusion criteria for the systematic review were (1) a longitudinal cohort or case-control study design that did not require mortality data to ascertain outcome; (2) the exposure variable was either a history of cancer diagnosis (yes vs no) at study baseline (prevalent cancer) or a new incident cancer diagnosis during follow-up (incident cancer), with studies categorized as including all cancer types, breast cancer, prostate cancer, colorectal cancer, or nonmelanoma skin cancer (NMSC); (3) the comparison group comprised individuals with no cancer history prior to baseline or no cancer history prior to each follow-up assessment; and (4) the outcome variable was an incident AD or dementia diagnosis. We selected breast cancer, prostate cancer, colorectal cancer, and NMSC because they account for nearly 40% of all US cancer cases and have relatively favorable survival probabilities.^24^

We initially screened titles and abstracts to select articles meeting our inclusion criteria. The Methods and Results sections of selected articles were reviewed for final inclusion. One reviewer (M.O.-R.) performed the literature search and screening, and 2 reviewers (M.O.-R. and L.C.K.) independently performed data extraction. Disagreements were minor and resolved through discussion between reviewers. Studies were included in the meta-analysis if a measure of association (risk ratio [RR], hazard ratio [HR], incidence rate ratio [IRR], or odds ratio [OR]) and 95% CIs were available.

Data items extracted were (1) study design, country, and study start and end dates; (2) study population source, inclusion and exclusion criteria, recruitment methods, and sample size; (3) participant characteristics, including age, sex, educational level, and race/ethnicity; (4) methods of cancer and AD diagnosis ascertainment; (5) analytic strategy, including model covariates; and (6) measure of association between cancer and AD and 95% CI. When multiple estimates were reported (eg, if possible and probable AD were included as separate outcomes), we used the measure of association for the outcome corresponding to stronger certainty of AD diagnosis.

### Evaluation of Methodological Study Biases with Causal Diagrams

**Figure 1.**
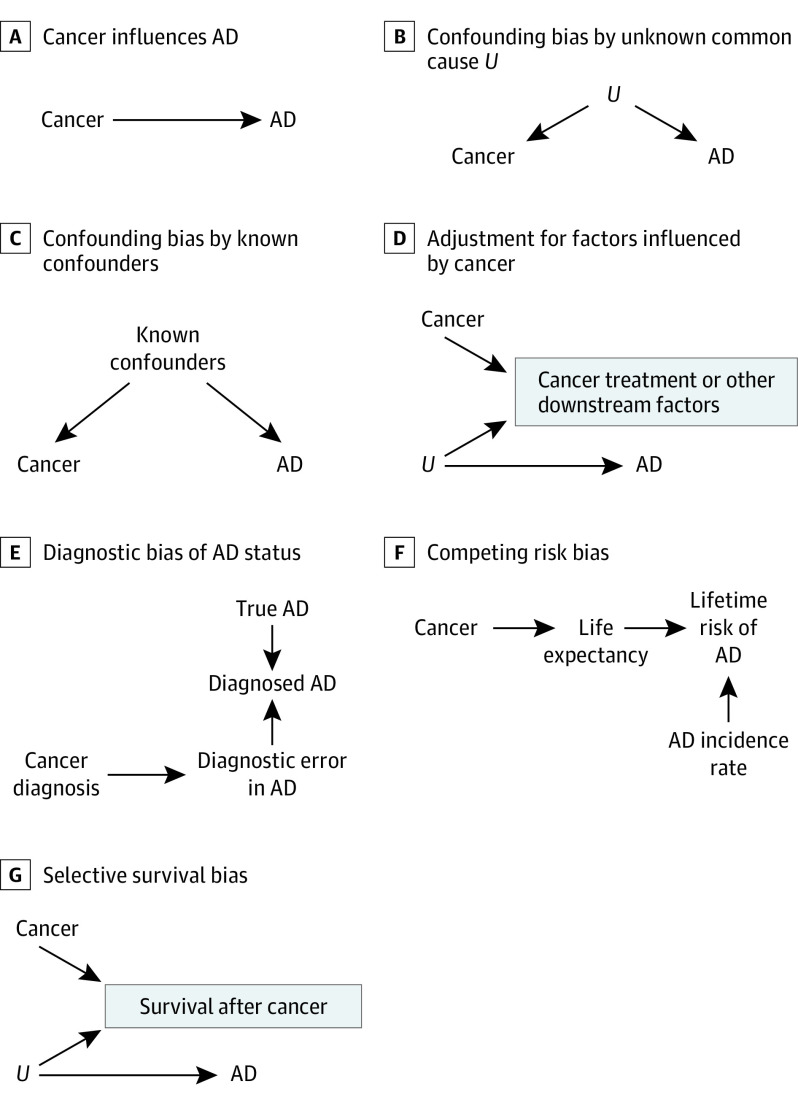
Directed Acyclic Graphs Depicting Alternative Explanations for the Observed Cancer–Alzheimer Disease (AD) Association The panel headings A through G correspond to the scenarios depicted in each panel. The directed acyclic graphs presented in panels A through G represent assumed data structures that could lead to spurious observed associations between cancer and AD. A, The direct arrow from cancer to AD indicates a causal association between cancer and subsequent AD risk. B, The direct arrows from unknown confounders *U* to cancer and to AD indicate that these conditions share a common cause. C-G, Alternative (noncausal) explanations for the cancer-AD association with no meaningful contribution of cancer to the etiology of neurodegeneration. C, The missing box around “Known confounders” indicates lack of statistical control for known confounders of the cancer-AD association. D, Adjustment for downstream variables, such as cancer treatment and comorbidities after cancer, is always inappropriate because it can introduce bias. E, A history of cancer diagnosis may influence the probability of receiving a diagnosis of AD. F, Cancer reduces life expectancy, and death is a competing risk to AD diagnosis. G, An unmeasured factor *U* promotes survival after cancer and reduces risk of AD. (The box around “Survival after cancer” indicates the restriction of the study population to those who survived cancer.)

We specified several causal structures that may account for the cancer-AD association, representing each with a directed acyclic graph (DAG)^25^ (Figure 1). Substantive interest in the cancer-AD association arises because of the possibility that cancer influences AD (Figure 1A) or that there is an unmeasured common cause of cancer and AD (Figure 1B). We considered alternative causal structures to be “biases” (Figures 1C-G). The DAGs illustrate how each type of bias could induce a spurious association between cancer and AD, which could be in either a negative (inverse) or a positive direction. Our appraisal of potential biases in the studies included in this review was informed by the causal structures in Figure 1.

Bias related to handling of confounders occurs when there are common causes of the exposure and outcome that are unaccounted for (Figures 1B and C). Confounders that would explain the observed inverse cancer-AD association would be those that raise risk of cancer but reduce risk of AD, ruling out many common lifestyle and social factors associated with increased risk of both conditions, such as smoking or alcohol consumption. We considered age, sex, and educational level as sociodemographic factors that should be included in a minimal adjustment set in all studies on this association. Another source of inappropriate statistical control is when models adjust for potential downstream consequences of cancer, including cancer treatment or comorbidities affected by cancer (Figure 1D). Adjustment for factors affected by the exposure of interest is a well-established source of bias.^26^ We considered studies that adjusted for age, sex, and educational level and those that did not adjust for downstream consequences of cancer as being less susceptible to bias owing to inappropriate handling of confounders.

Diagnostic bias of AD may occur if having a cancer diagnosis affects the probability of AD diagnosis (Figure 1E). Diagnostic bias can occur if clinicians are likely to overlook AD symptoms in patients receiving chemotherapy or other cancer treatments or if they are less likely to search for AD symptoms in patients with reduced life expectancy. Studies that ascertain AD from electronic health records have the greatest potential for diagnostic bias of AD. In Figure 1E, the arrow from cancer diagnosis to error in diagnosing AD represents this scenario. Diagnostic bias is avoided in studies in which all participants have an equal probability of AD diagnosis, independent of their cancer status, such as community-based cohorts in which cognition is routinely assessed at predetermined intervals for all participants.^27^ Diagnostic bias of cancer is also of interest because it could explain the inverse cancer-AD association if preexisting cognitive impairments lead to underdiagnosis or late diagnosis of cancer.^28,29^ We considered community-based studies and those that restricted their sample to participants who were cognitively intact at baseline as less susceptible to diagnostic bias.

Competing risks are events that preclude the occurrence of the primary outcome of interest.^30^ Here, death is a competing risk to AD, and a cancer diagnosis increases risk of death (Figure 1F). Studies that report cumulative incidence proportions of AD are subject to competing risks bias because the cumulative incidence proportion does not account for death. Cumulative incidences are usually estimated from unadjusted analyses, Kaplan-Meier curves, or regression models such as Fine and Gray.^31^ We considered longitudinal studies that used rate-based estimators such as HRs or IRRs^30^ or case-control studies that used incidence density sampling^32^ as having no competing risks bias.

Survival bias can occur if the study sample is enriched for individuals who have protective characteristics that promote cancer survival and reduce AD risk, which are usually unknown or unmeasured. Figure 1G illustrates this type of bias with the unknown AD protective characteristics denoted as *U.* Survival bias could arise even in longitudinal studies reporting HRs, because the cohort might become enriched over time for cancer survivors with unknown AD protective characteristics.^33^ Therefore, we considered studies on the association between NMSC and AD as less susceptible to survival bias because this type of cancer does not meaningfully raise mortality risk.^34^

Studies that include individuals with cancer diagnosis prior to baseline (ie, prevalent cancer cases) in their sample may be particularly vulnerable to survival bias because the sample will be limited to cancer survivors who did not receive a diagnosis of AD in the time frame from their diagnosis to the study baseline. This type of bias could be avoided by either excluding prevalent cancer cases from the analysis or providing separate estimates for prevalent and incident cancer cases. We considered studies that separated prevalent and incident cancer cases in the analysis as less susceptible to survival bias.

We also considered 2 other study characteristics that could lead to selection bias: (1) a high percentage (>5%) of missing covariate data, which decreases the analytic sample size, and, if not missing at random, may induce a bias similar to survival bias; and (2) studies with restrictive inclusion and exclusion criteria, such as including only participants with no comorbidities, which also can induce a bias similar to survival bias.

### Statistical Analysis

For the systematic review, we calculated the proportion of all studies reporting a positive or negative (inverse) statistically significant association between cancer and AD (*P* < .05). In the meta-analyses and metaregressions, the primary outcome was an RR, OR, IRR, or HR comparing AD risk between individuals with vs without a previous cancer diagnosis. Effect estimates were transformed to the natural log (ln RR, ln OR, ln IRR, and ln HR) for analyses, then back-transformed for interpretation.

To estimate the pooled association between cancer and AD, we conducted separate meta-analyses of cohort and case-control studies, combining all cancer types. Because some studies had subgroup analyses of cancer types, we additionally conducted meta-analyses stratified by cancer type. We first estimated fixed-effect models and assessed heterogeneity using the Higgins and Thompson *I^2^* statistic,^35^ followed by random-effects models. We used maximum likelihood to estimate between-study variance and the Knapp-Hartung method to estimate the variance of the summary effect estimate.^36^

Finally, we performed random-effects metaregressions to assess the influence of each type of methodological study bias on the pooled estimates. We developed 9 metaregression models in total, 1 model per type of study bias, adjusting for study design (case-control vs cohort) as a covariate. The metaregression coefficients represent the effects of each type of bias on the pooled cancer-AD risk estimate. Specifically, the metaregression coefficient is the difference in the pooled ln HR for cancer-AD association in studies with vs without the type of study bias being modeled. The intercepts from the metaregression models represent the pooled ln HR for cancer-AD association in studies without the type of study bias being modeled. Because we are interested in identifying types of study bias that could account for the observed inverse association between cancer and AD, metaregression coefficients that move the pooled HR away from the null value in the inverse direction indicate that the bias being modeled in that metaregression may contribute to the inverse cancer-AD association. We assessed publication bias with a funnel plot of standard error by ln HR. Analyses were conducted with R, version 3.6.3, using the Meta and Metafor packages (R Project for Statistical Computing), with a 2-sided *P* < .05 indicating statistical significance.

## Results

### Selection and Characteristics of Included Studies

Our search returned 2764 unique records; 22 studies (19 cohort studies and 3 case-control studies) met the eligibility criteria and were included (eFigure 1 in the Supplement). In total, 13 studies^10,37,38,39,40,41,42,43,44,45,46,47,48^ investigated all cancer types (eTable 2 in the Supplement); 5 studies^49,50,51,52,53^ investigated prostate cancer; 3 studies^9,54,55^ investigated NMSC; and 1 study^56^ investigated breast cancer (Table 1). Five studies that investigated all cancer types (5 of 13) additionally reported cancer type–specific subgroup analyses.

**Table 1.** Overview of the Studies Investigating the Association Between Cancer and AD by Cancer Type

Source	Study design	Country	Study Period	Study participants, No.	Age at baseline, y	Educational level	White participants
All cancer types							
Bowles et al,^10^ 2017	Population-based cohort study	US	1994-2005	4357 (42% men); 756 prevalent cancer; 583 incident cancer	Median, 75 (IQR, 70-80) prevalent cancer; median, 73 (IQR, 69-78) cancer-free	<College degree: 49% prevalent cancer; 49% cancer-free at baseline	92% prevalent cancer; 81% cancer-free at baseline
Driver et al,^40^ 2012	Population-based cohort study	US	1986-2008	1278 (39% men); 176 prevalent cancer; 247 incident cancer	Median, 77 (range, 68-96) prevalent cancer, 76 (range, 68-96) cancer-free	Completed secondary school: 72% prevalent cancer; 67% cancer-free at baseline	Not reported
Frain et al,^46^ 2017	US veteran cohort study	US	1996-2001	3 499 378 (98% men); 771 285 incident cancer	Median, 71 (IQR, 65-76) cancer group; median, 71 (IQR, 65-77) cancer-free group	Not reported	74% cancer group; 71% cancer-free group
Freedman et al,^41^ 2016	Cohort study of Medicare population in SEER regions	US	1992-2005	1 163 327 (50% men); 742 805 incident cancer	Median, 74 (range, 66-85) cancer group; median, 67 (range, 66-85) cancer-free group	Not reported	84% cancer-free group
Hanson et al,^45^ 2017	Population-based cohort study	US	1992-2009	92 425 (48% men); 2630 history of cancer and AD diagnosis	Range, 65-79	Not reported	Not reported
Musicco et al,^38^ 2013	Population-based cohort study	Italy	2004-2009	204 468 with cancer diagnosis (57% men)	Mean (SD), 72.4 (7.8)	Not reported	Not reported
Nudelman et al,^39^ 2014	Cross-sectional case-control study	US	2003	1609 (56% men); 313 AD cases; 1296 AD controls	Mean (SD) age range, 77-71 (5-8); reported by categories of AD and cancer status	Mean years education, range: 15-17 (SD, 2-3); reported by categories of AD and cancer status	Range, 81%-95%; reported by categories of AD and cancer status
Ording et al,^47^ 2020	Population-based cohort study	Denmark	1980-2013	949 309 cancer cases (48% men); 679 122 cases were 5:1 matched to cancer-free controls	Cancer cases: median, 83.1 (IQR, 77.9- 87.5); cancer-free controls: median, 83.5 (IQR: 78.6-87.7)	Not reported	Not reported
Prinelli et al,^48^ 2018	Population-based nested case-control study	Italy	1991-2012	1515 in the original cohort; 54 AD cases (56% men); 216 AD controls (age-, sex-, and smoking-matched 1:4)	Mean (SD), 62.1 (7.2)	≥Primary school: 61% AD cases; 67% AD controls	Not reported
Realmuto et al,^44^ 2012	Clinic-based case-control study	Italy	2006-2010	378 (29% men); 126 AD cases; 256 AD controls (age- and sex-matched)	Mean (SD), 76.7 (6.8) at interview; mean (SD), 71.1 (7.5) at AD diagnosis	>8 y of education: 18% AD cases; 29% AD controls	Not reported
Roe et al,^43^ 2005	Cohort study	US	1992-?^a^	249 (35% men); 50 with cancer history at baseline	Mean (SD), 78.1 (10.2) cancer group; mean (SD), 79.5 (9.8) cancer-free group	Mean (SD), y: 15 (2.9) cancer group; 14 (23.2) cancer-free group	100% cancer group; 94% cancer-free group
Roe et al,^37^ 2010	Population-based cohort study	US	1992-1999	3020 (41% men); 522 prevalent cancer; 376 incident cancer	Mean (SD), 75.9 (5.3) prevalent cancer; mean (SD), 74.9 (5.2) cancer-free	Mean (SD), y: 13 (3.3) prevalent cancer, 13 (3.2) cancer-free group	92% prevalent cancer; 90% cancer-free group
Sun et al,^42^ 2020	Population-based cohort study	Sweden	1992-2015	2 502 258 (55% men); 732 901 incident cancer	Median birth year 1931 in cancer group and cancer-free group	≥12 y: 18% cancer group; 17% cancer-free group	Not reported
PC							
Chung et al,^52^ 2016	Population-based cohort study	Taiwan	2001-2013	5340 men; 1335 incident PC; 4005 age-matched cancer-free men	Mean (SD), 72.2 (9.3)	Not reported	Not reported
Ng et al,^50^ 2018	Population-based cohort study	Australia	2003-2004	40 304 men; 3664 incident PC with ADT; age-matched (ratio 1:10) with cancer-free men	92% were ≥65	Not reported	Not reported
Robinson et al,^53^ 2018	Population-based cohort study	Sweden	2006-2014	146 985 men; 25 967 incident PC cases; year of birth– and county-matched (ratio 1:5) cancer-free men	Mean (SD), 76.5 (7.6)	High educational level: 18% cancer group; 18% cancer-free group	Not reported
Shahinian et al,^51^ 2006	Cohort study of Medicare population in SEER regions	US	1992-2001	101 089 men; 50 613 incident PC between 1992-1997	>66; median age, 72 in cancer-free group and cancer without ADT (75 for ADT group)	In zip code area with >12 y of education: 77% cancer group without ADT; 77% cancer-free group	In zip code area: 83% cancer group without ADT; 84% cancer-free group
Smith et al,^49^ 2018	Medicare inpatient hospital or skilled nursing facility cohort study	US	1986-1997	549 525 men; 115 189 with PC	PC: mean (SD), 70.4 (2.41); cancer-free group not reported	Not reported	100%
NMSC							
Schmidt et al,^9^ 2017	Population-based cohort study	Denmark	1980-2013	1 297 318 (49% men); 216 221 incident NMSC	Median, 68 (IQR, 58-78)	Not reported	Not reported
White et al,^55^ 2013	Population-based cohort study	US	1993-2009	1102 (42% men); 109 prevalent NMSC; 32 incident NMSC	Mean (SD), 79.4 (5.1) prevalent cancer; mean (SD), 78.0 (4.8) incident cancer; mean (SD), 78.9 (5.5) cancer-free group	Mean (SD), y: 15.1 (3.3) prevalent cancer; 13.4 (4.2) incident cancer; 13.2 (3.5) cancer-free group	97% prevalent cancer; 100% incident cancer; 67% cancer-free group
Wu et al,^54^ 2011	Cohort study	US	2003-2009	241 534 (56% men); 120 767 with NMSC; age-, sex-, region-, and calendar year–matched (ratio 1:1) to cancer-free people	Mean, 76.4	Not reported	Not reported
BC							
Sun et al,^56^ 2016	Population-based cohort study	Taiwan	2000-2004	120 985 women; 24 197 incident BC; age- and index year–matched (ratio 1:4) cancer-free women	BC: mean (SE), 49.5 (0.04); cancer-free group; mean (SE), 49.6 (0.07)	Not reported	Not reported

Abbreviations: AD, Alzheimer disease; ADT, androgen deprivation therapy; BC, breast cancer; IQR, interquartile range; NMSC, nonmelanoma skin cancer; PC, prostate cancer; SEER, Surveillance, Epidemiology, and End Results program of the National Cancer Institute.

^a^ Final calendar year of follow-up not reported.

Studies used different methods to ascertain cancer and AD diagnostic statuses. Cancer diagnoses were identified by self-report during study interviews (6 studies), linked data from cancer registries or surveillance systems (11 studies), and claims data from hospitals, ambulatory centers, or pharmacies (7 studies) (eTable 1 in the Supplement). The AD diagnoses were ascertained using claim codes in electronic health records in more than half the studies (15 studies). Seven studies ascertained AD diagnostic status through direct clinical assessments of study participants and through histopathology (Table 2).

**Table 2.** Study Methods of AD Diagnosis Ascertainment

Method and criteria	Source
Direct within-study assessments of participants	
The National Institute of Neurological and Communicative Disorders and Stroke–Alzheimer Disease and Related Disorders Association criteria	Bowles et al,^10^ 2017; Driver et al,^40^ 2012; Nudelman et al,^39^ 2014; Realmuto et al,^44^ 2012; Roe et al,^43^ 2005; Roe et al,^37^ 2010; White et al,^55^ 2013
Cognitive testing to evaluate multiple domains of cognitive function	Bowles et al,^10^ 2017; Driver et al,^40^ 2012; Nudelman et al,^39^ 2014
Histopathology	Roe et al,^43^ 2005
Electronic health records	
Medical claims using *ICD-9* code 331.0 (AD diagnosis)	Chung et al,^52^ 2016; Frain et al,^46^ 2017; Freedman et al,^41^ 2016; Hanson et al,^45^ 2017; Prinelli et al,^48^ 2018; Musicco et al,^38^ 2013; Shahinian et al,^51^ 2006; Smith et al,^49^ 2018; Sun et al,^56^ 2016; Wu et al,^54^ 2011
Medical claims using *ICD-9* code 290.0 (dementia, senile), 290.21 (with depressive features), 290.3 (acute confusional state)	Frain et al,^46^ 2017; Shahinian et al,^51^ 2006; Smith et al,^49^ 2018; Sun et al,^56^ 2016; Sun et al,^42^ 2020
Medical claims using *ICD-9* code 294 (other organic chronic psychotic conditions), 297 (delusional disorders), 310 (specific nonpsychotic mental disorders due to organic brain damage)	Smith et al,^49^ 2018; Sun et al,^56^ 2016
Medical claims with *ICD-10* codes (G30-G30.9-AD diagnosis)	Ording et al,^47^ 2020; Prinelli et al,^48^ 2018; Robinson et al,^53^ 2018; Schmidt et al,^9^ 2017; Sun et al,^42^ 2020
Medical claims with *ICD-8* codes	Ording et al,^47^ 2020; Schmidt et al,^9^ 2017
Pharmacy claims for donepezil, rivastigmine, galantamine, memantine	Chung et al,^52^ 2016; Musicco et al,^38^ 2013; Ng et al,^50^ 2018; Prinelli et al,^48^ 2018; Robinson et al,^53^ 2018
Mortality registry, exemption code for AD	Musicco et al,^38^ 2013; Prinelli et al,^48^ 2018

Abbreviations: AD, Alzheimer disease; *ICD-8*, *International Classification of Diseases, Eighth Revision*; *ICD-9*, *International Classification of Diseases, Ninth Revision*; *ICD-10*, *International Statistical Classification of Diseases and Related Health Problems, Tenth Revision*.

### Overview of Study Results and Meta-analysis

**Figure 2.**
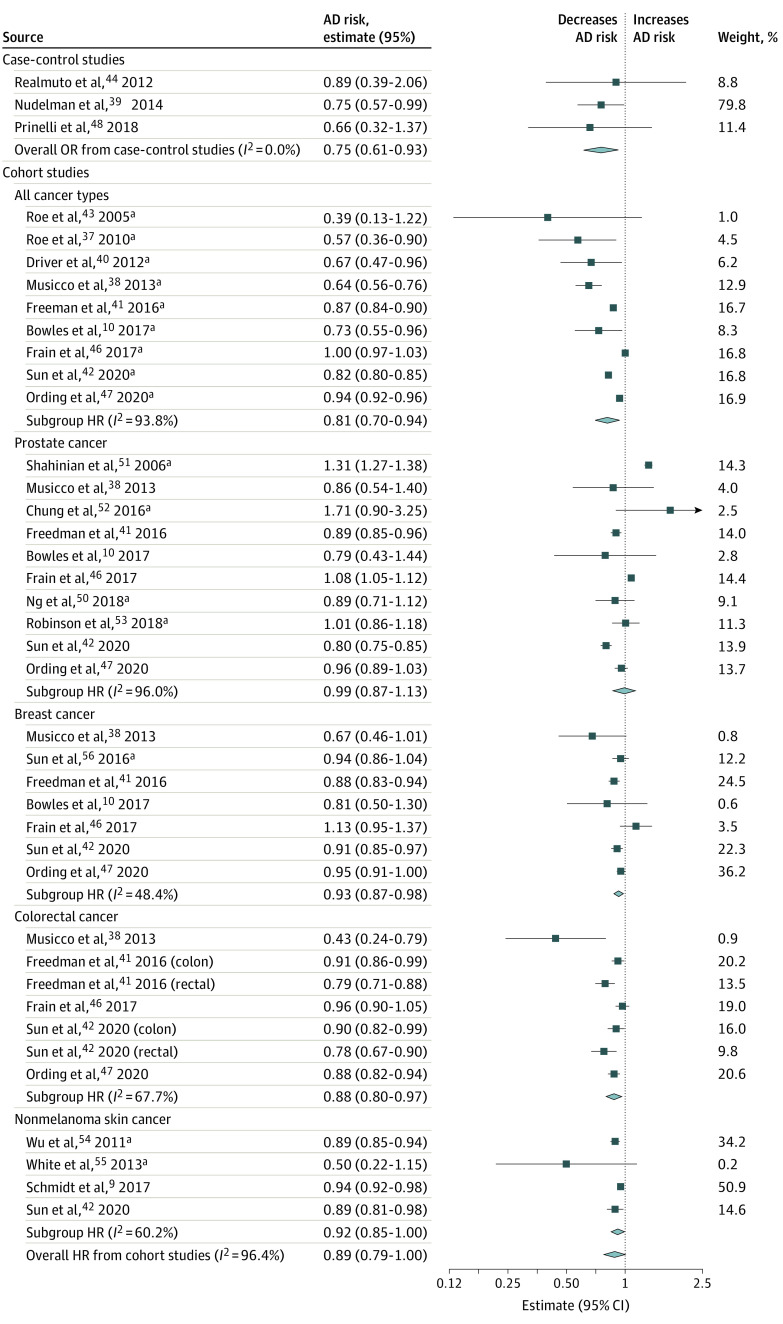
Forest Plot of Random-Effects Models for the Pooled Cancer–Alzheimer Disease (AD) Risk Estimates^a^ Random-effects meta-analyses were stratified by cancer type and study design. HR indicates hazard ratio; OR, odds ratio. Solid squares represent individual study estimates. The diamonds represent pooled estimates from the random-effects models. ^a^The random-effects meta-analysis for cohort studies (16 studies) includes only the main study results to avoid double counting study participants when cancer type-specific subgroup analyses were performed.

Overall, 11 of 22 included studies^9,10,37,38,39,40,41,42,47,49,54^ (50%) observed a statistically significant inverse association between cancer and AD, and 7 of 22 studies^43,44,45,50,55,56,57^ (32%) observed an inverse association that was nonstatistically significant. Two studies^51,52^ observed positive associations between cancer and AD, and 2 other studies^46,53^ found effectively null results. In the meta-analysis of all cohort studies (16 studies), the pooled fixed-effect HR for AD in cancer survivors compared with people with no cancer history was 0.94 (95% CI, 0.93-0.95). The *I*^2^ statistic was 96.4%, indicating high heterogeneity. The corresponding random-effects model provided a summary HR of 0.89 (95% CI, 0.79-1.00) (Figure 2). The random-effects OR for case-control studies was 0.75 (95% CI, 0.61-0.93) (Figure 2).

Heterogeneity was observed in stratified meta-analyses of cohort studies using all types of cancer (*I*^2^* = *93.8%) and prostate cancer (*I*^2^* = *96.0%), with less heterogeneity in analyses of breast cancer (*I*^2^* = *48.4%), colorectal cancer (*I*^2^* = *67.7%), and NMSC (*I*^2^* = *60.2%). The meta-analysis of case-control studies using all types of cancer did not exhibit heterogeneity (*I*^2^* = *0.0%). Because heterogeneity was substantial in most meta-analyses, we interpret the random-effects models as the primary findings (Figure 2).

Most studies had at least 1 type of bias, but the estimated pooled HR remained in the inverse direction when accounting for each of these biases in the metaregressions (Table 3). Details on specific biases by study are shown in eTable 3 in the Supplement). The most common biases were survival bias (cancer type that raises subsequent mortality risk; 20 studies), diagnostic bias (cancer status might influence AD ascertainment; 15 studies), and confounding bias (missing adjustment for at least 1 key sociodemographic factor; 120 studies). In studies subject to AD diagnostic bias, the cancer-AD HR was 0.94 (95% CI, 0.58-1.52), which was closer to the null value than in studies not susceptible to AD diagnostic bias (HR, 0.73; 95% CI, 0.58-0.90) (Table 3). The AD diagnostic bias accounted for 16.7% of the between-study variance in cancer-AD risk estimates. The effects of survival and confounding biases were small and accounted for less than 10% of between-study variance in cancer-AD risk estimates (Table 3).

**Table 3.** Overview of Methodological Study Biases

Estimate	Types of methodological study biases								
	Bias from handling of potential confounders		Diagnostic bias		Competing risks, estimated cumulative risks	Survival bias and related biases			
	Missing adjustment for age, sex, or educational level	Adjusted for factors influenced by cancer	Cognitively impaired individuals not excluded at baseline	Cancer status might influence AD diagnosis		Prevalent cancers not separated from incident cancers	Cancer type that raises subsequent mortality risk	High % of missing data	Restrictive inclusion and exclusion criteria
Studies with bias, No.	12	4	5	15	6	6	20	3	1
Metaregression estimates^a^									
Pooled ln HR (95% CI) in studies without the bias	−0.15 (−0.34 to 0.04)	−0.15 (−0.28 to −0.02)	−0.09 (−0.22 to 0.03)	−0.32 (−0.54 to −0.10)	−0.13 (−0.26 to 0.00)	−0.09 (−0.20 to 0.02)	−0.19 (−0.57 to 0.20)	−0.10 (−0.22 to 0.01)	−0.12 (−0.24 to 0.00)
Difference in ln HR (95% CI) for studies with the bias	0.04 (−0.20 to 0.29)	0.13 (−0.13 to 0.39)	−0.14 (−0.45 to 0.16)	0.26 (0.01 to 0.52)	0.09 (−0.32 to 0.50)	−0.34 (−0.71 to 0.03)	0.07 (−0.33 to 0.48)	−0.46 (−1.13 to 0.22)	−0.01 (−0.92 to 0.90)
*R*^2^, %	1.6	32.4	22.1	16.7	6.7	21.1	5.5	16.3	6.2

Abbreviations: AD, Alzheimer disease; ln HR, natural log hazard ratio.

^a^ Metaregressions adjusted for study design (case-control vs cohort) as a covariate.

Less common types of bias included inappropriate adjustment for downstream consequences of cancer, which were most commonly cancer treatments^46,51^ or comorbidities after cancer^37^ (4 studies); prevalent cancers not separated from incident cancers (4 studies); and individuals with cognitive impairment not being excluded at baseline (3 studies). Studies with inappropriate adjustment for downstream consequences of cancer had a mean HR of 0.98 (95% CI, 0.66-1.48), closer to the null value than studies without this bias (HR, 0.86; 95% CI, 0.76-0.99). Studies that combined prevalent and incident cancers had a mean HR farther from the null value (HR, 0.65; 95% CI, 0.40-1.05) than studies that did not combine prevalent and incident cancers (HR, 0.91; 95% CI, 0.82-1.02) (Table 3). Studies that did not exclude individuals with cognitive impairment at baseline had estimates farther from the null value than studies that excluded these individuals (Table 3). We observed an association between study sample size and the magnitude of the cancer-AD risk estimate, suggestive of publication bias (eFigure 2 in the Supplement).

## Discussion

In this systematic review and meta-analysis, we observed that individuals with history of cancer had a mean 11% lower risk of AD than those with no cancer history.^7,8^ We conducted metaregressions to quantify the directions and magnitudes of the effects of study biases on the pooled cancer-AD risk estimates. We found that biases due to inappropriate handling of potential confounders, diagnostic bias, and competing risks bias were unlikely to explain the inverse cancer-AD association. However, survival bias remains a possible explanation for the inverse cancer-AD association.

Our results align with earlier meta-analyses, which reported approximately 35% lower AD risk in cancer survivors than in those with no cancer history.^7,8^ Our evaluation of study biases sheds light on whether current evidence is sufficient to rule out methodological study biases as explanations for the cancer-AD association. Surprisingly, the studies most susceptible to diagnostic bias of AD status influenced the pooled cancer-AD risk estimate toward the null value, which suggests that diagnostic bias is an unlikely explanation for the observed inverse association.^20^ People with cancer history may be more likely to receive an AD diagnosis than cancer-free individuals owing to increased surveillance and detection through increased interaction with the health care system.^6^ Our results indicate that survival bias may contribute to the observed inverse cancer-AD association.

The best evidence against survival bias is from 4 studies of NMSC, a cancer with high survival rates. Another strategy to address survival bias is to report HRs stratified by time since cancer diagnosis because bias from survival should increase with more time elapsed since cancer diagnosis. Three studies^41,42,46^ included in this review presented time-varying HRs, but evidence from these reports is inconclusive.

### Strengths and Limitations

This review adhered to PRISMA guidelines, and we systematically quantified methodological biases that may spuriously explain the association under study. We integrated evidence from observational studies using different methodological approaches to understand the cancer-AD association. We did not consider all study designs that might be relevant to understanding this association, such as mendelian randomization,^58,59^ negative control studies, and studies with proxy measurements of AD (such as longitudinal decline of cognitive function,^11^ neuroimaging,^39^ or other functional assessments of the brain).^60^ Furthermore, many studies in this review were subject to multiple methodological biases; however, the number of studies was too small to allow evaluation of the impact of multiple biases simultaneously.

## Conclusions

This study found a weak inverse association between cancer and AD that does not appear to be explained by bias related to handling of confounders, diagnostic bias, or competing risks. Integrating results from different methodological approaches to this research question increases confidence that cancer and AD may share a common causal factor, potentially offering a novel path to understanding the shared etiologies of carcinogenesis and neurodegeneration. Survival bias cannot yet be ruled out as an explanation. Further studies designed to minimize survival bias are necessary to help determine whether survival or a true common etiology between cancer and AD explains the observed association.

## Notes

Supplement. **eFigure 1.** PRISMA Flow Diagram of Screening and Inclusion Process **eTable 1.** Study Methods of Cancer Diagnosis Ascertainment **eTable 2.** Cancer Types Reported in Studies in the Category “All Cancer Types” **eTable 3.** Overview of Methodological Study Biases **eFigure 2.** Funnel Plot of Study Standard Error (a Function of Sample Size) by lnHR for Longitudinal Cohort Studies Estimating HRs for AD Risk (*k* = 16)
