# Supplementary material for: Association Between Alzheimer Disease and Cancer With Evaluation of Study Biases: A Systematic Review and Meta-analysis
Source: JAMA Netw Open. 2020 Nov 13;3(11):e2025515. doi: 10.1001/jamanetworkopen.2020.25515 (PMC7666424; doi:10.1001/jamanetworkopen.2020.25515)
Supplement: Supplement. — eFigure 1. PRISMA Flow Diagram of Screening and Inclusion Process eTable 1. Study Methods of Cancer Diagnosis Ascertainment eTable 2. Cancer Types Reported in Studies in the Category “All Cancer Types” eTable 3. Overview of Methodological Study Biases eFigure 2. Funnel Plot of Study Standard Error (a Function of Sample Size) by lnHR for Longitudinal Cohort Studies Estimating HRs for AD Risk (k = 16) [file jamanetwopen-e2025515-s001.pdf]

Supplementary Online Content

Ospina-Romero M, Glymour MM, Hayes-Larson E, et al. Association between Alzheimer disease and cancer with evaluation of study biases: a systematic review and meta-analysis incorporating evaluation of study biases. *JAMA Netw Open*. 2020;3(11):e2025515. doi:10.1001/jamanetworkopen.2020.25515

eFigure 1. PRISMA Flow Diagram of Screening and Inclusion Process

eTable 1. Study Methods of Cancer Diagnosis Ascertainment

eTable 2. Cancer Types Reported in Studies in the Category "All Cancer Types"

eTable 3. Overview of Methodological Study Biases

eFigure 2. Funnel Plot of Study Standard Error (a Function of Sample Size) by lnHR for Longitudinal Cohort Studies Estimating HRs for AD Risk ($k = 16$)

This supplementary material has been provided by the authors to give readers additional information about their work.

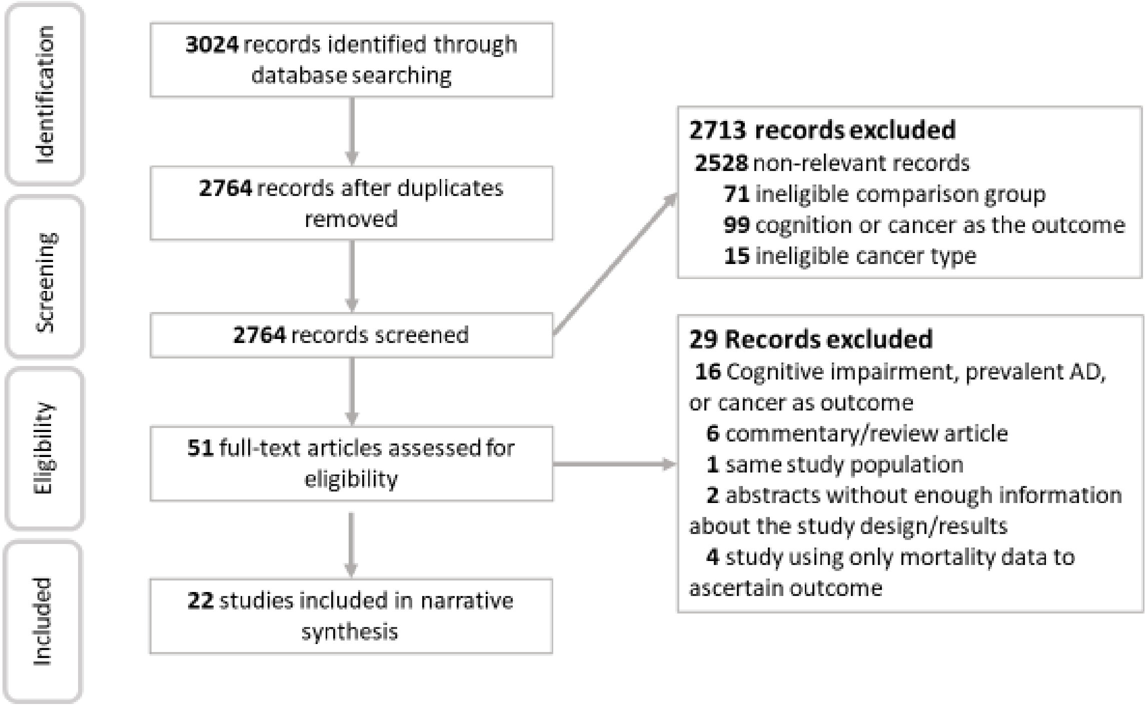

eFigure 1. PRISMA Flow Diagram of Screening and Inclusion Process. Studies considered ineligible cancer type were studies that investigated one type of cancer and this cancer was not breast, prostate, colorectal, or non-melanoma skin cancer.

© 2020 Ospina-Romero M et al. *JAMA Network Open*.

eTable 1. Study Methods of Cancer Diagnosis Ascertainment

Method of ascertainment	Defined in the analysis as:	Study
Self-reported cancer	Three groups: prevalent, time-varying incident cancer, and no cancer during follow-up.	White 2013
	Two groups: Cancer (prevalent and incident cases) and no cancer. Cancer variable was updated to include new cases during follow-up.	Driver 2012
	Two groups: Prevalent cancer and no cancer at baseline	Roe 2005
		Roe 2010
	Two groups: history of cancer or no history of cancer at the time of AD diagnosis	Nudelman 2014
		Realmuto 2012
Linking data from cancer registries or surveillance research programs (e.g. Surveillance, Epidemiology, and End Result (SEER) program)	Three groups: prevalent cancer, time-varying incident cancer, and no cancer. Prevalent cancer cases assigned to incident cancer group if they had a new malignancy during follow-up.	Bowles 2017
	Two groups: Cancer (prevalent and incident combined) and no cancer. Cancer variable was updated to include new cases during follow-up.	Driver 2012
	Multiple approaches to define cancer groups. Authors aimed to demonstrate biases introduced by these analytical approaches.	Hanson 2017
	Two groups: Incident cancer and random sample of cancer-free controls.	Musicco 2013
		Ording 2020
		Prinelli 2018
		Robinson 2018
		Shahinian 2006
		Schmidt 2017
		Sun 2020
		Freedman 2016
Medical claims (hospitals, ambulatory centers, procedures)	New cancer diagnosis, recurrent primary or metastatic cancer (Roe 2010).	Frain 2017
		Roe 2010
		Chung 2016

© 2020 Ospina-Romero M et al. *JAMA Network Open*.

		Wu 2011
		Sun 2016
		Smith 2018
Pharmacy claims	First dispensed use of androgen deprivation therapy for prostate cancer vs no cancer controls.	Ng 2018

© 2020 Ospina-Romero M et al. *JAMA Network Open*.

eTable 2. Cancer Types Reported in Studies in the Category "All Cancer Types"

Study	Cancer types
Bowles et al. 2017	Oral cavity/pharynx, colon and rectum, other digestive system, lungs and bronchus, soft tissue including heart, skin, breast, female genital system, prostate, urinary system, lymphoma.
Driver et al. 2012	Head and neck, esophagus or stomach, colon, rectum, pancreas, lung, hematological, connective tissue, melanoma, breast, uterus and endometrium, cervix, ovary, prostate, kidney, brain lymph nodes, other.
Frain et al. 2017	Prostate, lung, colorectal, breast, bladder, melanoma, lymphoma, leukemia, renal, myeloma, esophagus, pancreas, stomach, other
Freedman et al. 2016	Oral cavity, esophageal, stomach, small intestine, colon, rectum, pancreas, larynx, lung and bronchus, melanoma, breast, cervix, uterus, ovary, prostate, bladder, kidney/renal pelvis, thyroid, leukemia,
Hanson et al. 2017	Non-malignant neoplasms and non-melanoma skin cancer were excluded.
Musicco et al. 2013	Breast, prostate, colon, lung, urinary bladder, gastric, metastases with/without unspecified primary tumor, rectal, liver, pancreas, kidney, lymphomas, uterine body, leukemias, multiple myeloma, brain, biliary system, larynx, ovary, other.
Nudelman et al. 2014	Breast, female other types, gastrointestinal, bladder, renal, oral cavity, glandular, leukemia, lymphoma, melanoma, non-melanoma skin cancer, prostate, male other types, other.
Ording et al 2020	Bladder, brain, breast, colon, kidney, leukemia, lung, melanoma skin cancer, non-melanoma skin cancer, pancreatic, prostate, others.
Prinelli et al. 2018	Included cancer sites/types were not reported
Realmuto et al 2012	Prostate, intestines, ovary, uterus, breast, skin, central nervous system, others
Roe et al. 2005	Included cancer sites/types were not reported
Roe et al 2010	Included cancer sites/types were not reported
Sun et al. 2020	Oral cavity, salivary gland, esophageal, stomach, small intestine, colon, rectum, anus, liver, pancreas, nose, lung, breast, cervix, endometrium, ovary, other female genitals, prostate, testis, other male genitals, kidney, urinary bladder, melanoma, skin, eye, nervous system, thyroid gland, endocrine glands, connective tissue, non-Hodgkin lymphoma, Hodgkin lymphoma, myeloma, leukemia

© 2020 Ospina-Romero M et al. *JAMA Network Open*.

eTable 3. Overview of Methodological Study Biases

	Types of Methodological Study Biases								
	Bias from handling of potential confounders		Diagnostic bias		Competing risks	Survival bias and related biases			
	Missing adjustment for age, sex, or education	Adjusted for factors influenced by cancer	Cognitively impaired individuals not excluded at baseline	Cancer status might influence AD diagnosis	Estimated cumulative risks (as opposed to incidence or hazard rates)	Prevalent cancers not separated from incident cancers	Cancer type that raises subsequent mortality risk	High % of missing data	Restrictive inclusion and exclusion criteria
Meta-regressions [a]									
Pooled lnHR (95% CI) in studies without the bias	-0.15 (-0.34, 0.04)	-0.15 (-0.28, -0.02)	-0.09 (-0.22, 0.03)	-0.32 (-0.54, -0.10)	-0.13 (-0.26, 0.00)	-0.09 (-0.20, 0.02)	-0.19 (-0.57, 0.20)	-0.10 (-0.22, 0.01)	-0.12 (-0.24, 0.00)
Difference in lnHR (95% CI) for studies with the bias	0.04 (-0.20, 0.29)	0.13 (-0.13, 0.39)	-0.14 (-0.45, 0.16)	0.26 (0.01, 0.52)	0.09 (-0.32, 0.50)	-0.34 (-0.71, 0.03)	0.07 (-0.33, 0.48)	-0.46 (-1.13, 0.22)	-0.01 (-0.92, 0.90)
R^2	1.6%	32.4%	22.1%	16.7%	6.7%	21.1%	5.5%	16.3%	6.2%
Studies of all cancer types									
Bowles et al. 2017									
Driver et al. 2012									
Frain et al. 2017									
Freedman et al. 2016									
Hanson et al. 2017[b]									
Musicco et al. 2013									
Nudelman et al. 2014				*					
Ording et al. 2020									
Prinelli et al. 2018									
Realmuto et al. 2012				*					
Roe et al. 2005									
Roe et al. 2010									
Sadahiro et al. 2019									
Sun et al. 2020									

© 2020 Ospina-Romero M et al. *JAMA Network Open*.

Yarchoan et al. 2017									
Studies of prostate cancer									
Chung et al. 2016									
Ng et al. 2018									
Robinson et al. 2018									
Shahinian et al. 2006									
Smith et al. 2018[b]									
Studies of nonmelanoma skin cancer									
Schmidt et al. 2017[c]									
White et al. 2013									
Wu et al. 2011									
Studies of breast cancer									
Sun et al. 2016					**				

[a] Meta-regressions additionally adjusted for study design (case-control vs cohort) as a covariate

[b] Studies not included in meta-regression because only age-stratified measures of association were reported

[c] Study not included in meta-regression to prevent double counting people from Denmark

* Case-control studies in which AD status might influence cancer ascertainment

**Estimated HR from Cox regression with Lunn-McNeil approach that incorporates competing risks

Abbreviations: AD, Alzheimer's disease; CI, Confidence interval

© 2020 Ospina-Romero M et al. *JAMA Network Open*.

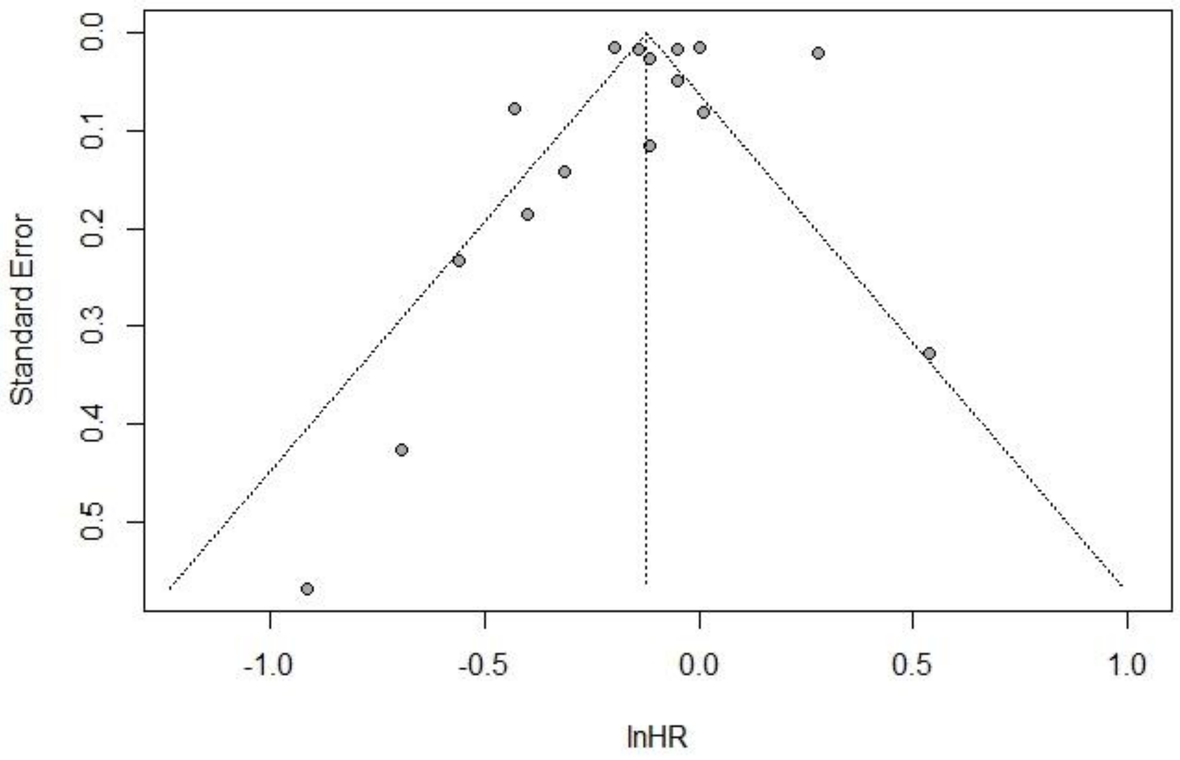

eFigure 2. Funnel Plot of Study Standard Error (a Function of Sample Size) by lnHR for Longitudinal Cohort Studies Estimating HRs for AD Risk (*k* = 16).

© 2020 Ospina-Romero M et al. *JAMA Network Open*.
